# Anesthetic Management Using Remimazolam for Transcatheter Edge-to-Edge Repair of the Mitral Valve in Patients With Reduced Ejection Fraction: A Case Report of Two Cases

**DOI:** 10.7759/cureus.30706

**Published:** 2022-10-26

**Authors:** Hisakatsu Ito, Akiyo Kameyama, Minako Furuta, Masashi Yoshida, Kenta Onishi, Masaaki Kawakami

**Affiliations:** 1 Anesthesiology, Toyama University Hospital, Toyama, JPN; 2 Anesthesiology, University of Toyama, Toyama, JPN; 3 Anesthesiology, Toyama Prefectural Central Hospital, Toyama, JPN

**Keywords:** mitral valve regurgitation, transcatheter edge-to-edge repair, vascular resistance, cardiac index, remimazolam

## Abstract

Remimazolam is an ultrashort-acting benzodiazepine that causes minimal hemodynamic changes. We present two patients, with reduced ejection fraction, who underwent remimazolam anesthesia for transcatheter edge-to-edge repair of the mitral valve with the MitraClip system. In case 1, the patient’s vitals were stable throughout the surgery. However, in case 2, which had a lower cardiac output, the patient’s blood pressure decreased remarkably after anesthesia induction. Though remimazolam does not alter the cardiac output, it reportedly has vasodilatory effects. Since remimazolam can reduce blood pressure in patients where the reduction in cardiac output is compensated for by high peripheral vascular resistance, caution should be exercised.

## Introduction

Remimazolam is an ultrashort-acting benzodiazepine that causes hemodynamic changes [1,2]. Successful anesthesia with remimazolam has been reported in several patients with impaired cardiac function [3,4]. In the two cases we are reporting, remimazolam was utilized for the induction and maintenance of anesthesia in patients with mitral regurgitation (MR) and reduced ejection fraction during transcatheter edge-to-edge repair of the mitral valve with the MitraClip system. The patients’ cardiac index (CI) and systemic vascular resistance index (SVRI), in addition to basic vital signs, were assessed. The usefulness and precautions of remimazolam are discussed in this report. The patients provided written consent for the publication of this case report.

## Case presentation

Case 1

An 83-year-old man (height, 160 cm; weight, 62 kg) with MR exacerbation and heart failure was scheduled for a MitraClipR (Abbott, Chicago, USA) procedure. Twenty years prior to the consultation, the patient underwent coronary artery bypass graft surgery for acute myocardial infarction. He was presented to our institution due to the development of acute heart failure secondary to the development of new onset coronary artery stenosis and severe MR after coronary intervention at the primary hospital. After a slight improvement in heart failure (New York Heart Association Class II) status due to medical therapy, transthoracic echocardiography revealed moderate functional MR (effective regurgitant orifice area of 0.19 cm^2^, regurgitant volume of 30 mL) with low ejection of 32%, and left ventricular diffuse hypokinesis and dilatation (end-diastolic diameter of 57 mm). The results of the preoperative pulmonary artery catheterization were a CI of 2.5 L/min/m^2^, mean pulmonary artery pressure of 23 mmHg, and a SvO_2_ of 63.5%. The patient’s medical history included chronic atrial fibrillation, hypertension, diabetes, chronic kidney disease (estimated glomerular filtration rate of 43.1 mL/min/1.73 m^2^), and dyslipidemia.

After establishing the arterial pressure-based cardiac output (FloTrac; Edwards Life Sciences, CA, USA) and bispectral index (BIS), in addition to standard monitoring, general anesthesia was induced by intravenous remimazolam (6 mg/kg/h), remifentanil (0.3 μg/kg/min), and continuous administration of noradrenaline (0.03 μg/kg/ min), to avoid undesirable vasodilation. After losing consciousness, remimazolam was adjusted to 0.1 to 1.0 mg/kg/h to maintain the BIS between 40 and 60. Rocuronium 50 mg was administered prior to tracheal intubation. No additional temporary vasopressors were administered. The key hemodynamic parameters during surgery are summarized in Table 1. A mean blood pressure of >60 mmHg was maintained after anesthesia induction. The CI before anesthesia induction was 3.0 L/min/m^2^, and it was hardly changed at a median of 2.9 L/min/m^2^ with a quartile range of 2.9-3.0 between induction and the end of surgery. After anesthesia induction, the systemic vascular resistance decreased and remained low until the end of surgery. The patient was quickly aroused two minutes after the administration of 0.25 mg of flumazenil. His consciousness was clear, and he could respond to the movements instructed. The next day, he was discharged from the cardiac care unit in good general condition with a delirium score of the Intensive Care Delirium Screening Checklist (ICDSC).

**Table 1:** Key hemodynamic parameters during surgery in case 1. The lowest value after induction is the lowest value within 30 minutes after the start of remimazolam administration. *It was calculated to refer to CVP values immediately after central venous catheter insertion. mBP: mean blood pressure, CVP: central venous pressure, CI: cardiac index, SVRI: systemic vascular resistance index.

Case 1	Before induction	Lowest value after induction	Start of surgery	Clip release	End of surgery
mBP (mmHg)	80	63	68	64	71
CVP (mmHg)	-	8	7	10	11
CI (L/min/m^2^)	3.0	2.9	3.0	2.9	2.9
SVRI (Dyn s cm^−5 ^m^2^)	1920*	1517	1627	1490	1655
ScvO_2_ (%)	-	70	70	79	79
BIS	92	42	46	43	60

Case 2

An 82-year-old woman (height, 162 cm; weight, 58 kg) with severe MR and chronic heart failure (New York Heart Association Class III) was scheduled for a MitraClipR procedure. Eleven years prior to the consultation, she was diagnosed with dilated cardiomyopathy. Transthoracic echocardiography revealed severe functional MR (effective regurgitant orifice area of 0.44 cm^2^, regurgitant volume of 53 mL) with a very low ejection of 24%, and left ventricular diffuse hypokinesis and dilatation (end-diastolic diameter of 57 mm). The preoperative pulmonary artery catheter had a CI of 1.8 L/min/m^2^, a mean pulmonary artery pressure of 31 mmHg, and an SvO_2_ of 51.4%. Her past medical history of paroxysmal atrial fibrillation, hypertension, and chronic kidney disease (estimated glomerular filtration rate of 11.0 mL/min/1.73 m^2^).

After establishing the FloTrac and BIS monitor, in addition to standard monitoring, remimazolam (6 mg/kg/h), remifentanil (0.3 μg/kg/min), and noradrenaline (0.03 μg/kg/min) were administered as similar to case 1, and rocuronium 50 mg was administered pre-intubation. Key hemodynamic parameters are summarized in Table 2. Prior to anesthesia induction, her CI was low at 1.4 L/min/m^2^, but her blood pressure was maintained due to high vascular resistance. Post-anesthesia induction, her mean blood pressure was significantly reduced, but the CI was generally maintained at a median of 1.9 L/min/m^2^ with a quartile range of 1.8-2.1 between induction and the end of the surgery, which suggests that the blood pressure reduction was due to vasodilation with the synergistic effect of remimazolam with remifentanil, and not due to impaired venous return from positive pressure ventilation after intubation. Stable circulatory dynamics were achieved by increasing noradrenaline levels and maintaining adequate vascular resistance. She was quickly aroused within four minutes after administering 0.25 mg of flumazenil. Her consciousness was clear, and she responded to the instructed movements. The next day, she was discharged from the cardiac care unit in good general condition with an ICDSC of 0.

**Table 2:** Key hemodynamic parameters during surgery in case 2. The lowest value after induction is the lowest value within 30 minutes after the start of remimazolam administration. *It was calculated to refer to CVP values immediately after central venous catheter insertion. mBP: mean blood pressure, CVP: central venous pressure, CI: cardiac index, SVRI: systemic vascular resistance index.

Case 2	Before Induction	Lowest value after induction	Start of surgery	Clip release	Clip release	End of surgery
mBP (mmHg)	68	33	60	53	57	57
CVP (mmHg)	-	7	10	-	11	11
CI (L/min/m^2^)	1.4	1.7	1.9	2.1	2.1	2.1
SVRI (Dyn s cm^−5 ^m^2^)	3485*	1223	2105	-	1752	1752
ScvO_2_ (%)	-	64	66	81	78	66
BIS	87	46	41	42	42	40

## Discussion

Remimazolam reportedly causes less circulatory depression than other general anesthetics [1,2]. A small randomized control trial reported that anesthesia induction with remimazolam during cardiac surgery was associated with fewer fluctuations in mean blood pressure [5].

This pharmacological feature is caused by the ability of remimazolam not to alter the cardiac output. Because vasopressors increase left ventricular pressure and regurgitant volume, maintaining cardiac output is crucial in the anesthesia of patients with valve regurgitant diseases such as MR. Thus, in cases of valve regurgitation, remimazolam should be utilized.

In the first case, the patient’s blood pressure was maintained after anesthesia induction, similar to previous case reports [3]. However, in case 2, the patient’s blood pressure markedly decreased after anesthesia induction. The patient in case 2 had an initially low CI, and her blood pressure was possibly maintained as compensation for high vascular resistance. Under such conditions, remimazolam could pose a risk of hypotension due to its vasodilating effects. This vasodilator effect may then need to be compensated for by increasing the amount of vasopressor medications or reducing the rate of remimazolam if allowed based on BIS values. The BIS value was low enough after anesthesia induction in case 2, which suggests induction at an even lower rate of remimazolam may have been safer. Preclinical research in sheep has reported that remimazolam causes low blood pressure due to dose-dependent vasodilatation [6].

Delirium is an independent risk factor for major postoperative complications [7], and benzodiazepines are reported to be associated with delirium risk [8]. It remains unclear whether remimazolam contributes to delirium similarly to benzodiazepines. Clinical research on the impact of remimazolam on postoperative delirium is still ongoing [9]. In both cases, the commonly used delirium assessment score, ICDSC, was 0 [10]. Thus, they were diagnosed with no symptoms suspicious of delirium.

## Conclusions

Remimazolam may be used in the anesthetic management of patients with low cardiac function. Moreover, it is significantly useful in patients with diseases associated with valve regurgitation. However, since remimazolam can cause severe hypotension in conditions with compensated circulation due to its high vascular resistance, caution should be applied in these cases.
